# Supplementary material for: High potency of sequential therapy with only β-lactam antibiotics
Source: eLife. 2021 Jul 28;10:e68876. doi: 10.7554/eLife.68876 (PMC8456660; doi:10.7554/eLife.68876)
Supplement: Supplementary file 1. — (A) List of antibiotics used for the evolution experiments. (B) Statistical analysis of main evolution treatments for the evolutionary dynamics shown in Figure 2A. (C) Statistical analysis of main evolution treatments for the multidrug β-lactam resistance after transfer 12 in Figure 2B. (D) Statistical analysis of main evolution treatments for the multidrug β-lactam resistance after transfer 48 in Figure 2C. (E) Statistical analysis of main evolution treatments for Shannon diversity in Figure 2B, C. (F) Comparison of resistance profiles between transfer 12 and transfer 48 for Figure 2—figure supplement 4 using Wilcoxon test and Bonferroni correctiona. (G) Minimum inhibitory concentrations (MICs) as determined by agar dilution for the three β-lactams and as used for the fluctuation assays shown in Figure 4. (H) Likelihood ratio test to assess pairwise variation in spontaneous resistance rates for the three β-lactam antibiotics shown in Figure 4B. (I) Post hoc comparisons based on the false discovery rate for phenotype of cross-resistance on secondary antibiotic as shown in Figure 4C. (J) Analysis of variance of the consequences of the main treatment type on the three measured responses for transfer 12 of the triple β-lactam experiment as summarized in Figure 5. (K) Post hoc comparison based on the false discovery rate of the effect of the main treatment types on extinction for the triple β-lactam experiment as summarized in Figure 5. (L) General linear model analysis of the consequences of the experimental predictors switching rate and irregularity on the three measured responses for transfer 12 of the triple β-lactam experiment as summarized in Figure 5. (M) Main effect tests for the consequences of switching rate and irregularity on extinction and multidrug resistance (MDR) for transfer 12 of the triple β-lactam evolution experiment as summarized in Figure 5. (N) General linear model analysis of the consequences of the three considered biological predictors, cumula [file elife-68876-supp1.docx]

**High potency of sequential therapy with only β-lactam antibiotics**

Aditi Batra*^1,2^, Roderich Roemhild*^1,2,3^, Emilie Rousseau^4^, Soeren Franzenburg^5^, Stefan Niemann^4^, and Hinrich Schulenburg^#,1,2^

**Supplementary File 1**

**Supplementary File 1A. List of antibiotics used for the evolution experiments**

| **Abbr.** | **Antibiotic** | **Supplier** | **Solvent** | **Stock, mg/ml** | **Powder, °C** |
| --- | --- | --- | --- | --- | --- |
| **β-lactam antibiotics (BL)** | | | | | |
| **AZL** | azlocillin | Sigma, A7926-1G  Prepare fresh | H_2_O | 50 | 4 |
| **CAR** | carbenicillin | Carl Roth, 6344.2  Prepare fresh | 50% ethanol | 50 | 4 |
| **CEF** | cefsulodin | Carl Roth, 4014.2  Light sensitive! | H_2_O | 20 | -20 |
| **CTZ** | ceftazidime | Sigma, C3809.1G  with 10% NaCO_3_  renew at least monthly | H_2_O (pH ~ 9.0) | 11  (= 10 mg/ml CTZ) | 4 |
| **DOR** | doripenem | Sigma, 32138-25MG | H_2_O (37°C) | 25 | 4 |
| **TIC** | ticarcillin | Sigma, T5639-1G  Prepare fresh | H_2_O | 100 | 4 |
| **Aminoglycosides (AG)** | | | | | |
| **GEN** | gentamicin solution | Roth, 2475.1 | Ready made | 50 | n.a. |
| **STR** | streptomycin | Sigma, S6501-5 | H_2_O | 25 | 4 |
|  |  |  |  |  |  |
| **Fluoroquinolones (FQ)** | | | | | |
| **CIP** | ciprofloxacin | Sigma, 17850-5G-F | 0.1 M HCl | 25 | RT |

**Supplementary File 1B. Statistical analysis of main evolution treatments for the evolutionary dynamics shown in Figure 2A^a^.**

| **Comparison** | ***z*** | ***P*** |
| --- | --- | --- |
| **Early phase (transfers 2-12)** |  |  |
| mono-fast regular | 3.16 | **0.00947** |
| mono-slow regular | 0.724 | 0.46897 |
| mono-random | 1.527 | 0.19012 |
| fast regular-slow regular | -2.505 | **0.03673** |
| fast regular-random | -1.948 | 0.10294 |
| slow regular-random | 0.76 | 0.46897 |
| **Middle phase (transfers 13-48)** |  |  |
| mono-fast regular | 1.407 | 0.319 |
| mono-slow regular | -0.779 | 0.523 |
| mono-random | 0.01 | 0.992 |
| fast regular-slow regular | -2.061 | 0.236 |
| fast regular-random | -1.444 | 0.319 |
| slow regular-random | 0.816 | 0.523 |
| **Late phase (transfers 49-96)** |  |  |
| mono-fast regular | 0.162 | 0.871 |
| mono-slow regular | -0.647 | 0.621 |
| mono-random | -1.399 | 0.507 |
| fast regular-slow regular | -0.723 | 0.621 |
| fast regular-random | -1.375 | 0.507 |
| slow regular-random | -0.685 | 0.621 |

^a^ The analysis was performed with R version 3.5.0. Posthoc pairwise comparisons based on *z* statistics, following analysis of a general linear mixed model (GLMM), including relative growth yield as the response variable and sequence and transfer as fixed factors and preculture and replicate population as nested random factors. Representation of the model using R package *nlme*: *lme(data=data_noex, rgr ~ evoexp_ID + transfer, random=list(~1|preculture, ~transfer|line)).* The defined model provided a better fit than a minimal model for all three phases (*Likelihood ratio*>254, p<0.0001). Significant probabilities are shown in bold (p-values were corrected for multiple testing using false discovery rate). Extinct lineages excluded.

**Supplementary File 1C. Statistical analysis of main evolution treatments for the multidrug β-lactam resistance after transfer 12 in Figure 2B^a^.**

| **Comparison** | ***z*** | ***P*** |
| --- | --- | --- |
| **Early phase (transfer 12)** |  |  |
| mono-fast regular | -11.715 | **< 2e-16** |
| mono-slow regular | -5.132 | **3.44E-07** |
| mono-random | -5.405 | **9.70E-08** |
| fast regular-slow regular | 6.355 | **4.18E-10** |
| fast regular-random | 8.004 | **3.33E-15** |
| slow regular-random | 0.521 | 0.602 |

^a^ The analysis was performed with R version 3.5.0. Posthoc pairwise comparisons based on *z* statistics, following analysis of a general linear mixed model (GLMM), including multidrug β-lactam resistance as the response variable and sequence as fixed factors and replicate population as nested random factors. Representation of the model using R package *lme4*: *lmer(data=dfm12, res_abc ~ evoexp_ID + (1|line)).* Significant probabilities are shown in bold (p-values were corrected for multiple testing using false discovery rate). Extinct lineages excluded.

**Supplementary File 1D. Statistical analysis of main evolution treatments for the multidrug β-lactam resistance after transfer 48 in Figure 2C^a^.**

| **Comparison** | ***z*** | ***P*** |
| --- | --- | --- |
| **Middle phase (transfer 48)** |  |  |
| mono-fast regular | -6.933 | **2.46E-11** |
| mono-slow regular | -2.763 | **0.00686** |
| mono-random | -2.77 | **0.00686** |
| fast regular-slow regular | 4.034 | **0.00011** |
| fast regular-random | 5.193 | **6.22E-07** |
| slow regular-random | 0.426 | 0.66996 |

^a^ The analysis was performed with R version 3.5.0. Posthoc pairwise comparisons based on *z* statistics, following analysis of a general linear mixed model (GLMM), including multidrug β-lactam resistance as the response variable and sequence as fixed factors and replicate population as nested random factors. Representation of the model using R package *lme4*: *lmer(data=dfm48, res_abc ~ evoexp_ID + (1|line)).* All significant p-values are shown in bold (p-values were corrected for multiple testing using false discovery rate). Extinct lineages excluded.

**Suppementary File 1E. Statistical analysis of main evolution treatments for Shannon diversity in Figs. 2B and 2C^a^.**

| **Variable** | **Mean squares** | ***F_1,4_*** | ***P*** |
| --- | --- | --- | --- |
| Main treatment type | 0.0656 | 0.7388 | 0.57349 |
| Transfer | 0.5513 | 6.206 | **0.01893** |

^a^ The analysis of variance for the response variable within-population Shannon diversity *H* was performed with R version 3.5.0. Significant probabilities are given in bold.

**Supplementary File 1F. Comparison of resistance profiles between transfer 12 and transfer 48 for Figure 2-figure supplement 4 using Wilcoxon test and Bonferroni correction^a^**

| Treatment | Antibiotic | n | *W* | *P* value | Adj. *P* value (Bonferroni) |
| --- | --- | --- | --- | --- | --- |
| 1 | CAR | 20 | 220 | 0.59782821 | 1 |
| 2 | CAR | 20 | 153 | 0.208389525 | 1 |
| 3 | CAR | 20 | 160 | 0.285191716 | 1 |
| 4 | CAR | 20 | 159.5 | 0.279183285 | 1 |
| 5 | CAR | 20 | 381 | 1.05E-06 | **8.37E-05** |
| 6 | CAR | 20 | 278 | 0.036030896 | 1 |
| 7 | CAR | 40 | 1309.5 | 9.68E-07 | **7.75E-05** |
| 8 | CAR | 20 | 340.5 | 0.000152034 | **0.012162728** |
| 9 | CAR | 20 | 37 | 1.10E-05 | **0.000877722** |
| 10 | CAR | 20 | 308.5 | 0.00091365 | 0.07309204 |
| 11 | CAR | 20 | 305.5 | 0.004494519 | 0.359561553 |
| 12 | CAR | 20 | 180.5 | 0.607252052 | 1 |
| 13 | CAR | 20 | 182 | 0.635897028 | 1 |
| 14 | CAR | 20 | 371 | 3.29E-07 | **2.63E-05** |
| 15 | CAR | 20 | 32 | 5.86E-06 | **0.000468941** |
| 16 | CAR | 20 | 367 | 6.67E-06 | **0.000533361** |
| 1 | CEF | 20 | 118 | 0.026329137 | 1 |
| 2 | CEF | 20 | 0 | 6.79E-08 | **5.43E-06** |
| 3 | CEF | 20 | 396 | 1.23E-07 | **9.86E-06** |
| 4 | CEF | 20 | 51 | 5.88E-05 | **0.004705435** |
| 5 | CEF | 20 | 231 | 0.413526312 | 1 |
| 6 | CEF | 20 | 0 | 6.77E-08 | **5.41E-06** |
| 7 | CEF | 40 | 1458 | 2.50E-10 | **2.00E-08** |
| 8 | CEF | 20 | 350.5 | 4.95E-05 | **0.003961378** |
| 9 | CEF | 20 | 92.5 | 0.003797686 | 0.303814881 |
| 10 | CEF | 20 | 348 | 9.61E-06 | **0.000768627** |
| 11 | CEF | 20 | 65 | 0.000274147 | **0.021931781** |
| 12 | CEF | 20 | 8 | 9.72E-10 | **7.78E-08** |
| 13 | CEF | 20 | 381.5 | 9.75E-07 | **7.80E-05** |
| 14 | CEF | 20 | 94 | 0.003533771 | 0.282701679 |
| 15 | CEF | 20 | 155 | 0.228628859 | 1 |
| 16 | CEF | 20 | 242.5 | 0.25589085 | 1 |
| 1 | CIP | 20 | 38.5 | 1.33E-05 | **0.001061249** |
| 2 | CIP | 20 | 60.5 | 0.000169102 | **0.013528148** |
| 3 | CIP | 20 | 266 | 0.076237748 | 1 |
| 4 | CIP | 20 | 112.5 | 0.018577116 | 1 |
| 5 | CIP | 20 | 359 | 1.80E-05 | **0.001442037** |
| 6 | CIP | 20 | 310 | 0.003049738 | 0.243979033 |
| 7 | CIP | 40 | 803 | 0.980807169 | 1 |
| 8 | CIP | 20 | 256 | 0.133228552 | 1 |
| 9 | CIP | 20 | 229.5 | 0.432666654 | 1 |
| 10 | CIP | 20 | 258 | 0.05785721 | 1 |
| 11 | CIP | 20 | 210.5 | 0.786667495 | 1 |
| 12 | CIP | 20 | 25 | 2.34E-06 | **0.000187429** |
| 13 | CIP | 20 | 157 | 0.250185682 | 1 |
| 14 | CIP | 20 | 77 | 0.000919379 | 0.073550297 |
| 15 | CIP | 20 | 321 | 0.001111142 | 0.08889139 |
| 16 | CIP | 20 | 138.5 | 0.098836627 | 1 |
| 1 | DOR | 20 | 255.5 | 0.136760312 | 1 |
| 2 | DOR | 20 | 0 | 6.76E-08 | **5.41E-06** |
| 3 | DOR | 20 | 376 | 2.05E-06 | **0.000163842** |
| 4 | DOR | 20 | 40 | 1.59E-05 | **0.001272761** |
| 5 | DOR | 20 | 302 | 0.00603322 | 0.482657568 |
| 6 | DOR | 20 | 0 | 6.77E-08 | **5.41E-06** |
| 7 | DOR | 40 | 1321 | 5.48E-07 | **4.38E-05** |
| 8 | DOR | 20 | 397 | 1.02E-10 | **8.12E-09** |
| 9 | DOR | 20 | 8 | 2.21E-07 | **1.76E-05** |
| 10 | DOR | 20 | 61 | 0.000151954 | **0.012156325** |
| 11 | DOR | 20 | 382.5 | 8.48E-07 | **6.78E-05** |
| 12 | DOR | 20 | 0 | 6.77E-08 | **5.41E-06** |
| 13 | DOR | 20 | 400 | 6.79E-08 | **5.43E-06** |
| 14 | DOR | 20 | 219 | 0.616758169 | 1 |
| 15 | DOR | 20 | 36 | 9.74E-06 | **0.000779089** |
| 16 | DOR | 20 | 288.5 | 0.017277768 | 1 |
| 1 | GEN | 20 | 331 | 0.000413167 | **0.033053323** |
| 2 | GEN | 20 | 240 | 0.285259522 | 1 |
| 3 | GEN | 20 | 317 | 0.001623613 | 0.129889034 |
| 4 | GEN | 20 | 318.5 | 0.001410423 | 0.112833831 |
| 5 | GEN | 20 | 305 | 0.004692804 | 0.375424326 |
| 6 | GEN | 20 | 301.5 | 0.006284199 | 0.502735906 |
| 7 | GEN | 40 | 901.5 | 0.331091086 | 1 |
| 8 | GEN | 20 | 366 | 7.57E-06 | **0.000605595** |
| 9 | GEN | 20 | 110.5 | 0.016054079 | 1 |
| 10 | GEN | 20 | 291.5 | 0.004535653 | 0.362852227 |
| 11 | GEN | 20 | 83 | 0.001622791 | 0.129823281 |
| 12 | GEN | 20 | 156.5 | 0.24472283 | 1 |
| 13 | GEN | 20 | 381 | 1.04E-06 | **8.33E-05** |
| 14 | GEN | 20 | 157 | 0.250274578 | 1 |
| 15 | GEN | 20 | 17.5 | 8.47E-07 | **6.77E-05** |
| 16 | GEN | 20 | 144.5 | 0.136760312 | 1 |

^a^ The analysis was performed with R version 3.6.3. Significant probabilities are given in bold. Source data are provided in Figure 2-source data 1.

**Supplementary File 1G. Minimum inhibitory concentrations (MICs) as determined by agar dilution for the three β-lactams and as used for the fluctuation assays shown in Figure 4.**

| **Antibiotic** | **MIC (mg/L)** |
| --- | --- |
| CAR | 50 |
| CEF | 1 |
| DOR | 0.06 |

**Supplementary File 1H. Likelihood Ratio Test to assess pairwise variation in spontaneous resistance rates for the three β-lactam antibiotics shown in Figure 4B^a^**

| Antibiotic 1 | Antibiotic 2 | *LR* | *P* |
| --- | --- | --- | --- |
| CAR | CEF | 260.68550 | **<0.0001** |
| CAR | DOR | 348.97050 | **<0.0001** |
| CEF | DOR | 10.01538 | **<0.01** |

^a^ The analysis was performed with the package *rSalvador* in R version 3.6.3. Significant probabilities are given in bold.

**Supplementary File 1I. Posthoc comparisons based on the false discovery rate for phenotype of cross-resistance on secondary antibiotic as shown in Figure 4C^a^**

| **Comparison** | ***P*** | **Adjusted *P*** |
| --- | --- | --- |
| DOR – CEF | **0.000218** | **0.000327** |
| DOR – CAR | **0.0000319** | **0.0000957** |
| CEF – CAR | 0.364 | 0.364 |

^a^ The analysis was performed with the package *rcompanion* in R version 3.6.3. Significant probabilities are given in bold.

**Supplementary File 1J. Analysis of variance of the consequences of the main treatment type on the three measured responses for transfer 12 of the triple β-lactam experiment as summarized in Figure 5^a^**

| **Response** | **Adjusted *R^2^*** | ***F_2,9_*** | ***P*** |
| --- | --- | --- | --- |
| Extinction | 0.63 | 10.19 | **0.0049** |
| Biomass increase | -0.22 | 0.003 | 0.997 |
| Multidrug resistance | 0.28 | 3.09 | 0.0953 |

^a^ The analysis was performed with R version 3.5.1. Separate analyses were performed for each response variable. The subscript for the *F* ratio indicates the degrees of freedom. The p-value gives the probability of the difference between a minimal and the defined model that includes the main treatment types as fixed factors. Significant probabilities are given in bold. The distribution of the response data is consistent with normality (Shapiro-Wilk test, *W*>0.89, p>0.15) and homogeneity of variance (Bartlett’s test, *κ^2^*<0.88, d.f.=2, p>0.64).

**Supplementary File 1K. Posthoc comparison based on the false discovery rate of the effect of the main treatment types on extinction for the triple β-lactam experiment as summarized in Figure 5^a^**

| **Comparison** | ***P*** |
| --- | --- |
| Fast regular – slow regular | **0.0076** |
| Fast regular – random | >0.99 |
| Slow regular – random | **0.0064** |

^a^ The analysis was performed with R version 3.5.1. The posthoc test was only performed for the response variable, for which the main treatment type had a significant effect (see above table). Significant probabilities are given in bold.

**Supplementary File 1L. General linear model analysis of the consequences of the experimental predictors switching rate and irregularity on the three measured responses for transfer 12 of the triple β-lactam experiment as summarized in Figure 5^a^**

| **Response** | **Adjusted *R^2^*** | ***F_2,9_*** | ***P*** |
| --- | --- | --- | --- |
| Extinction | 0.68 | 12.48 | **0.0025** |
| Biomass increase | -0.21 | 0.03 | 0.9667 |
| Multidrug resistance | 0.32 | 3.60 | 0.0712 |

^a^ The analysis was performed with R version 3.5.1. Separate analyses were performed for each response variable. The subscript for the *F* ratio indicates the degrees of freedom. The p-value gives the probability of the difference between a minimal and the defined model that includes switching rate and irregularity as fixed factors. Significant probabilities are given in bold.

**Supplementary File 1M. Main effect tests for the consequences of switching rate and irregularity on extinction and multidrug resistance (MDR) for transfer 12 of the triple β-lactam evolution experiment as summarized in Figure 5^a^**

|  | **Extinction** |  |  | **MDR** |  |
| --- | --- | --- | --- | --- | --- |
| **Factor** | ***F_1_*** | ***P*** |  | ***F_1_*** | ***P*** |
| Switching rate | 14.44 | **0.0042** |  | 3.0 | 0.1172 |
| Irregularity | 10.53 | **0.0101** |  | 4.19 | 0.0711 |

^a^ Effect tests were only performed for response variables, for which the GLM analysis revealed at least a statistical trend (p*<*0.1) of an improvement of the defined model over a minimal model, thus only including extinction and multidrug resistance (MDR). See above table for the results. The effect tests were performed with R version 3.5.1. The subscript of the *F* ratio denotes the degrees of freedom. Significant probabilities are given in bold.

**Supplementary File 1N. General linear model analysis of the consequences of the three considered biological predictors, cumulative probability of spontaneous resistance, cross-resistance, and hysteresis on the three measured responses for transfer 12 of the triple β-lactam experiment as summarized in Figure 5^a^**

| **Response** | **Adjusted *R^2^*** | ***F_3,8_*** | ***P*** |
| --- | --- | --- | --- |
| Extinction | 0.52 | 4.91 | **0.0320** |
| Biomass increase | -0.01 | 0.97 | 0.4516 |
| Multidrug resistance | 0.22 | 2.02 | 0.1899 |

^a^ The analysis was performed with R version 3.5.1. Separate analyses were performed for each response variable. The p-value gives the probability of the difference between a minimal and the defined model that includes cumulative probability of spontaneous resistance, cross-resistance, and cumulative hysteresis level as fixed factors. Significant probabilities are given in bold.

**Supplementary File 1O. Main effect tests for the consequences of the three considered biological predictors, cumulative probability of spontaneous resistance, cross-resistance, and hysteresis on extinction of the triple β-lactam evolution experiment as summarized in Figure 5^a^**

|  | **Extinction** |  |
| --- | --- | --- |
| **Factor** | ***F_1_*** | ***P*** |
| Spontaneous resistance | 4.14 | 0.0763 |
| Collateral effects | 10.42 | **0.0121** |
| Hysteresis | 0.16 | 0.7015 |

^a^ Effect tests were only performed for the response variable, for which the GLM analysis revealed a significant improvement of the defined model over a minimal model, thus only including extinction. See above table for the results. The effect tests were performed with R version 3.5.1. The subscript of the *F* ratio denotes the degrees of freedom. Significant probabilities are given in bold.
