# Supplementary material for: High potency of sequential therapy with only β-lactam antibiotics
Source: eLife. 2021 Jul 28;10:e68876. doi: 10.7554/eLife.68876 (PMC8456660; doi:10.7554/eLife.68876)
Supplement: Transparent reporting form [file elife-68876-transrepform.docx]

***eLife’s* transparent reporting form**

We encourage authors to provide detailed information *within their submission* to facilitate the interpretation and replication of experiments. Authors can upload supporting documentation to indicate the use of appropriate reporting guidelines for health-related research (see [EQUATOR Network](http://www.equator-network.org/%20)), life science research (see the [BioSharing Information Resource](https://biosharing.org/)), or the [ARRIVE guidelines](http://www.plosbiology.org/article/info:doi/10.1371/journal.pbio.1000412) for reporting work involving animal research. Where applicable, authors should refer to any relevant reporting standards documents in this form.

If you have any questions, please consult our Journal Policies and/or contact us: [editorial@elifesciences.org](mailto:editorial@elifesciences.org).

**Sample-size estimation**

- You should state whether an appropriate sample size was computed when the study was being designed
- You should state the statistical method of sample size computation and any required assumptions
- If no explicit power analysis was used, you should describe how you decided what sample (replicate) size (number) to use

Please outline where this information can be found within the submission (e.g., sections or figure legends), or explain why this information doesn’t apply to your submission:

We did not perform a specific power analysis. For each experiment, we aimed at a sample size, which was as high as possible while still allowing us to run all treatments and replicates in parallel and in randomized order.

**Replicates**

- You should report how often each experiment was performed
- You should include a definition of biological versus technical replication
- The data obtained should be provided and sufficient information should be provided to indicate the number of independent biological and/or technical replicates
- If you encountered any outliers, you should describe how these were handled
- Criteria for exclusion/inclusion of data should be clearly stated
- High-throughput sequence data should be uploaded before submission, with a private link for reviewers provided (these are available from both GEO and ArrayExpress)

Please outline where this information can be found within the submission (e.g., sections or figure legends), or explain why this information doesn’t apply to your submission:

A definition of biological versus technical replication is given in the methods section. There we also provide numbers of biological and technical replicates for each experiment and how often an experiment was performed. We included all relevant data in our analyses to address and statistically evaluate a particular question or hypothesis. We did not encounter extreme outliers, which would have required specific handling.

Whole genome sequencing data is available from NCBI under the BioProject number: PRJNA704789, as also indicated in the methods section of the manuscript. The read-only access for this data is:

<https://dataview.ncbi.nlm.nih.gov/object/PRJNA704789?reviewer=p8v6dusm5ei0160r5qvf94loha>

**Statistical reporting**

- Statistical analysis methods should be described and justified
- Raw data should be presented in figures whenever informative to do so (typically when N per group is less than 10)
- For each experiment, you should identify the statistical tests used, exact values of N, definitions of center, methods of multiple test correction, and dispersion and precision measures (e.g., mean, median, SD, SEM, confidence intervals; and, for the major substantive results, a measure of effect size (e.g., Pearson's r, Cohen's d)
- Report exact p-values wherever possible alongside the summary statistics and 95% confidence intervals. These should be reported for all key questions and not only when the p-value is less than 0.05.

Please outline where this information can be found within the submission (e.g., sections or figure legends), or explain why this information doesn’t apply to your submission:

Statistical methods are described in the last section of the methods section. Whenever we describe the results of statistical tests, then we provide information on the used test. All detailed results of the statistical evaluation are provided in supplementary tables, including the used tests, the test statistics, and also the method used for correction of multiple testing. Sample sizes are given in the methods, and also the figure legends. The raw data used for statistical analysis and generation of figures is provided in supplementary source data files.

(For large datasets, or papers with a very large number of statistical tests, you may upload a single table file with tests, Ns, etc., with reference to sections in the manuscript.)

**Group allocation**

- Indicate how samples were allocated into experimental groups (in the case of clinical studies, please specify allocation to treatment method); if randomization was used, please also state if restricted randomization was applied
- Indicate if masking was used during group allocation, data collection and/or data analysis

Please outline where this information can be found within the submission (e.g., sections or figure legends), or explain why this information doesn’t apply to your submission:

For our experiments, the relevant treatment groups were run in parallel and in randomized set-ups. Treatment names were masked, in order to minimize observer bias.

**Additional data files (“source data”)**

- We encourage you to upload relevant additional data files, such as numerical data that are represented as a graph in a figure, or as a summary table
- Where provided, these should be in the most useful format, and they can be uploaded as “Source data” files linked to a main figure or table
- Include model definition files including the full list of parameters used
- Include code used for data analysis (e.g., R, MatLab)
- Avoid stating that data files are “available upon request”

Please indicate the figures or tables for which source data files have been provided:

Source data is provided for all figures and statistical analyses.
